# Photo-activation of the delocalized lipophilic cation D112 potentiates cancer selective ROS production and apoptosis

**DOI:** 10.1038/cddis.2017.19

**Published:** 2017-02-02

**Authors:** Ning Yang, Michael Weinfeld, Hélène Lemieux, Ben Montpetit, Ing Swie Goping

**Affiliations:** 1 Department of Biochemistry, University of Alberta, Edmonton, Alberta T6G 2H7, Canada; 2 Department of Oncology, University of Alberta, Edmonton, Alberta T6G 2H7, Canada; 3 Faculty Saint-Jean, University of Alberta, Edmonton, Alberta T6G 2H7, Canada; 4 Cell Biology, University of Alberta, Edmonton, Alberta T6G 2H7, Canada

## Abstract

Delocalized lipophilic cations (DLCs) selectively accumulate in cancer cell mitochondria and have long been explored for therapeutic applications. Although targeted effects to cancer cells are demonstrated *in vitro*, non-specific toxicities *in vivo* have hampered clinical development. Identifying the molecular mechanisms of action and enhancing selectivity are thus necessary next steps to improve these compounds and evaluate their suitability for further drug development. D112 is one such DLC with promising properties. We previously demonstrated that D112 selectively induced intrinsic apoptosis in transformed *versus* non-transformed cell lines. Here we show that D112 preferentially entered transformed cells where it interacted with, and damaged mitochondrial DNA, inhibited Complex I respiration and induced reactive oxygen species (ROS). ROS production was critical for Bax activation and subsequent apoptosis. Importantly, photo-activation of D112 potentiated selective ROS production and increased the window of toxicity towards cancer cells over non-transformed cells. Thus photodynamic therapy would be an exciting adjunct to D112 studies and may be generally applicable for other DLCs that are currently under therapeutic investigation.

The small molecule D112^1^ belongs to a class of compounds known as delocalized lipophilic cations (DLCs). These compounds traverse hydrophobic plasma membranes, accumulate in mitochondria and trigger cell death.^2^ Based on their mitochondria-sensing ability, DLCs have been developed for numerous applications such as imaging, targeted drug delivery and therapeutic agents. As examples, fluorescent DLCs, such as MitoTracker Red and JC-1, are widely used as research tools for cell biology studies,^3, 4^ and the triphenylphosphine has been shown to direct chemotherapeutic agents to the mitochondria.^5, 6^ Relevant to our study, a number of DLCs display selective killing of carcinoma cells over normal cells, stimulating interest in their development as anti-cancer compounds.^7^

The cancer cell-selective toxicity of DLCs is attributed to the elevated plasma and/or mitochondrial membrane potentials of carcinoma cells.^2, 6^ Once DLCs enter the mitochondria, they cause mitochondrial dysfunction. Rhodamine 123 (Rh-123) was the first DLC to demonstrate toxicity to mitochondria *in vitro*^8, 9^ by impairing the F_0_F_1_-ATPase.^10, 11^ Dequalinium chloride (DECA) damaged mitochondrial DNA (mtDNA) and blocked the electron transport system (ETS) by inhibiting NADH-ubiquinone reductase.^12, 13^ MKT-077 perturbed mitochondrial membranes resulting in non-specific inhibition of respiratory system components.^14^ Further, the anti-cancer activity of MKT-077 was tested in animal models,^15, 16^ and led to subsequent clinical trials that were terminated due to renal toxicity.^17^ MKT-077 served as a prototype for the development of DLCs with improved specific toxicity.^18^ Thus, DLCs have demonstrated anti-cancer cell activity *in vitro* with potential for development into viable therapeutic options.

D112 is a photosensitizer that was developed by the Eastman Kodak Company for use in photographic emulsions and was subsequently found to have promising properties when assessed in a cancer drug-screening program of approximately 2000 structural dye variants.^19^ We identified that D112 induced cell death in carcinoma-derived cell lines to a greater extent than non-transformed cell lines, accumulated in mitochondria and induced apoptosis that was dependent on BAX/BAK and inhibited by Bcl-2.^1^ In the current study, we investigated the mechanisms of D112-induced cellular toxicity, selective cancer cell uptake and explored strategies to enhance cancer cell specific activity. We identified that mitochondrial respiration and reactive oxygen species (ROS) were critical for D112-toxicity. D112-mediated ROS production triggered Bax activation and subsequent apoptosis of cancer-cells. By exploiting the inherent fluorescent properties of D112, we discovered that photo-activation potentiated D112 cytotoxicity and increased the selective effects towards cancer-cells. Therefore a combination of D112 and photodynamic therapy (PDT) could be explored for potential applications against cancer.

## Results

### D112-induced cell death was enhanced by mitochondrial respiration

**Figure 1:**
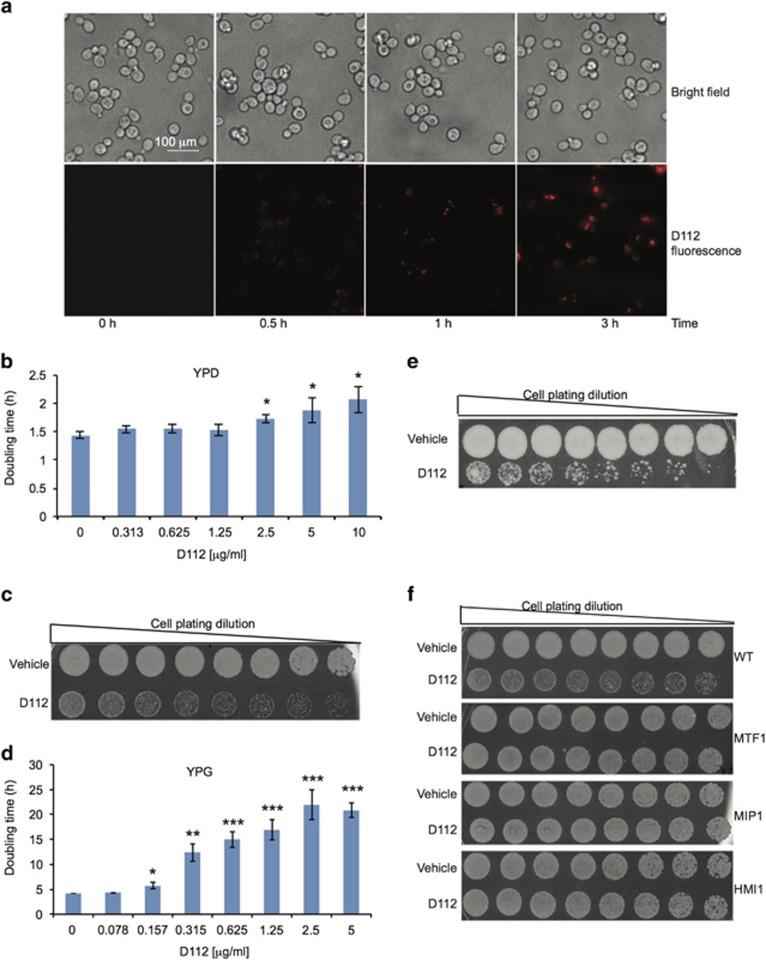
Effect of D112 treatment on yeast growth. (**a**) Yeast cells were incubated with 5 *μ*g/ml D112 for the indicated times followed by fluorescence microscopy. Shown are representative images of two independent experiments. Scale bar, 100 *μ*m. (**b**) Yeast doubling time in YPD. (**c**) Yeast viability following D112 treatment in YPD. (**d**) Yeast doubling time in YPG. (**e**) Yeast viability following D112 treatment in YPG. (**f**) Viability of indicated yeast mutants following 5 *μ*g/ml D112 treatment. All values represent the mean±S.D. of three independent experiments. *P<0.05, **P<0.01, ***P<0.001

To explore the contribution of mitochondria to D112-induced cytotoxicity, we employed *Saccharomyces cerevisiae* as a model system. We first verified that D112 was taken up by yeast (Figure 1a) and affected yeast growth (Supplementary Figure S1a). D112 decreased the yeast proliferative rate as demonstrated by a dose-dependent increase in doubling times (Figure 1b). To assess cell viability, we washed D112-treated cells in fresh media and either spotted bulk serial dilutions (Figure 1c) or plated equal cell number on YPD recovery plates lacking D112 (Supplementary Figure S1b). A four-fold reduction in colony viability confirmed that D112 induced yeast cell death (Supplementary Figure S1b).

Given that yeast utilize fermentative metabolism when cultured in standard glucose-containing medium (YPD as in Figures 1b and c), we switched growth to non-fermentable carbon medium (YPG-glycerol) that drives mitochondrial respiration. These conditions sensitized yeast cells to D112 with significant growth defects at concentrations that were 15-fold lower than the lowest effective dose in YPD (Figure 1d and Supplementary Figure S1c). Moreover, cell viability was reduced approximately 40-fold (Figure 1e and Supplementary Figure S1d) indicating that mitochondrial respiration sensitizes cells to D112-toxicity.

To confirm that mitochondrial respiration was required for D112-toxicity, we hypothesized that respiration-deficient mutants (petite strains) would be resistant. We tested this postulate using various mutant strains that lack gene(s) necessary for oxidative phosphorylation and rely on glycolytic fermentation for growth. We examined 11 rho^-^ petite strains that harbored single gene mutations in ETS components and F_0_F_1_ ATP synthase, as well as rho^0^ mtDNA-deleted mutants that are deficient for multiple genes for electron transport and mitochondrial metabolism (Supplementary Table S1). D112 inhibited cell proliferation in all mutant strains tested (Supplementary Figure S2a), indicating that the growth-arrest effect of D112 was not dependent on mtDNA or those single components of the ETS and F_0_F_1_ ATP synthase. In contrast, when assessed for cell viability, only the rho^0^ mutants that lack mtDNA (MTF1, MIP1 and HMI1) were protected from D112-induced cell death (Figure 1f and Supplementary Figure S2b). These results showed that D112 induced growth arrest independent of mtDNA and respiration, whereas mtDNA and/or its downstream gene products were necessary for cell death.

### D112 binds to DNA

**Figure 2:**
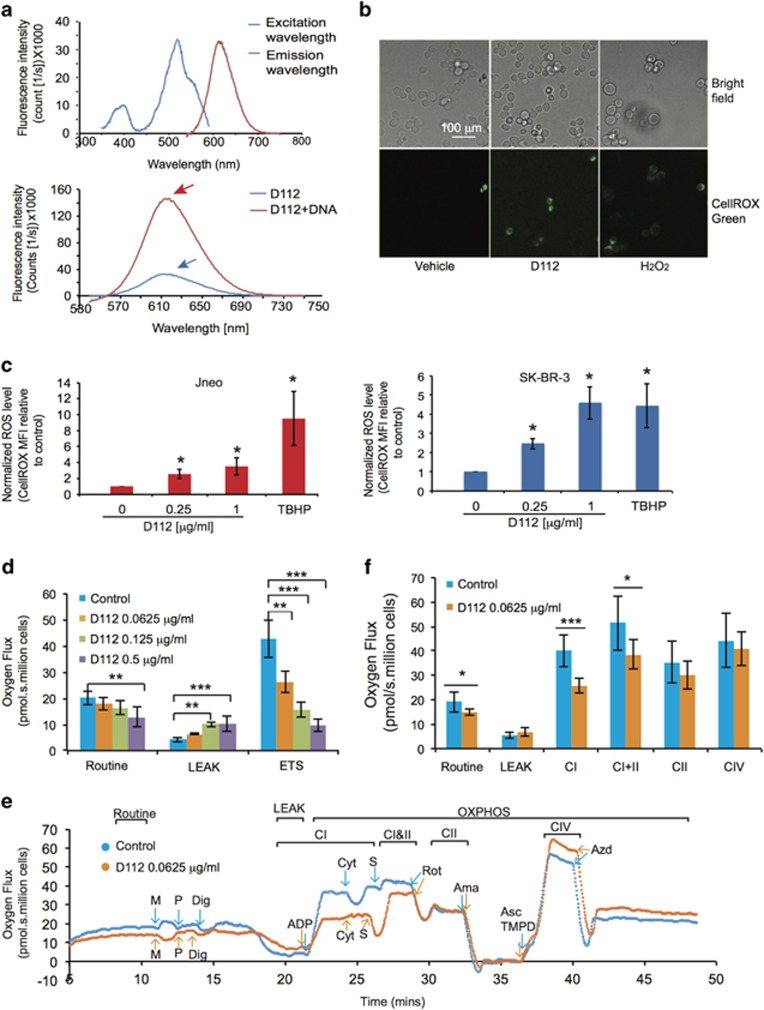
DNA-binding and ROS-inducing properties of D112. (**a**) (Top) Excitation and emission spectra of D112. (Bottom) Emission fluorescence intensity of D112 in the presence of DNA. (**b**) D112-induced ROS production in yeast as indicated by CellROX Green fluorescence. Scale bar, 100 *μ*m. (**c**) D112-induced ROS production in mammalian cell lines assessed by flow cytometry. Normalized ROS level was MFI of (treated cells/untreated cells). (**d**) Measurement of oxygen consumption in the presence of D112 at indicated concentrations. Routine: the cellular respiration in the presence of endogenous substrates. LEAK: resting respiration after Oligomycin addition to inhibit ATP synthase. ETS: maximal electron transport capacity determined by the stepwise addition of the uncoupler FCCP. (**e**) Representative trace of oxygen consumption (flux) in the absence or presence of D112 pretreatment of permeabilized cells. Arrows indicate time of titration of the substrates and inhibitors. Ama, antimycin a; Asc, ascorbate; Azd, sodium azide; CI, Complex I respiration; CI+II, Complex I and II respiration; CII, Complex II respiration; CIV, Complex IV respiration; Cyt, cytochrome c; Dig, digitone; M, malate; OXPHOS, oxidative phosphorylation; P, pyruvate; S, succinate; Rot, rotenone; TMPD, tetramethyl-phenylenediamine. (**f**) Quantification of oxygen flux from **e**. All values represent the mean±S.D. of three independent experiments. *P<0.05, **P<0.01, ***P<0.001

Based on the protective effect of rho^0^ strains, we assessed D112 binding to DNA using an electrophoretic gel-shift assay (Supplementary Figure S3a). D112 migrated partially into the gel when mixed with DNA. Further, since DNA-binding can alter emission intensity by changing the molecular environment of the fluorophore,^20^ we compared D112 fluorescence in the presence or absence of DNA. D112 alone had two excitation peaks (380-410 nm and 510-530 nm) and one emission peak at 615 nm (Figure 2a, top). In the presence of DNA, fluorescence emission intensity increased five-fold (Figure 2a, bottom, see arrows). This DNA-dependent fluorescent property of D112 is reminiscent of ethidium bromide which is also known to damage mtDNA and generate petite strains in yeast.^21^ We tested whether D112 induced the petite phenotype of the W303-1A yeast strain that grows as red colonies under normal conditions, but grows as white colonies when induced to become petites. D112 treatment induced the white-growing petite phenotype (Supplementary Figure S3b). Finally, we demonstrated that D112 induced mtDNA strand breaks through a PCR-based mtDNA double-strand break assay performed in mammalian cells (Supplementary Figures S3c and d). These data suggested that D112 bound to mtDNA, and induced mtDNA damage.

### ROS induction is required for D112-mediated cell death

While the loss of mtDNA and loss of D112-binding sites may contribute to D112-resistance of rho^0^ cells, the lack of mitochondrial-encoded genes could also contribute to the resistant phenotype. The mitochondrial genome encodes components of Complexes I, III, IV and the F_0_F_1_ ATP synthase.^22^ Complexes I and III produce ROS during respiration, and this capacity is lost in rho^0^ cells.^23, 24^ Since sudden rises of ROS induce cell death,^24, 25, 26, 27^ we explored whether D112 treatment increased ROS in wild-type yeast cells. Using the fluorescent indicator CellROX Green, we observed an increase in ROS levels in D112-treated cells (Figure 2b), suggesting that ROS production was involved in D112-mediated cell death. We expanded this finding in two human cell lines, Jneo (human Jurkat T lymphoblastoid cell line) and SK-BR-3 (human breast carcinoma), both of which are sensitive to D112.^1^ ROS was assessed with indicators CellROX Green (Figure 2c) or CM-H_2_DCFDA (Supplementary Figures S4a and b), and we confirmed that D112 significantly increased ROS levels in Jneo and SK-BR-3 cells (3- and 4.5-fold, respectively).

To examine whether D112 induced ROS production by inhibiting electron flow, we used high-resolution respirometry to measure mitochondrial oxygen consumption in Jneo cells. Incubation with increasing amounts of D112 caused a decrease in Routine respiration (Figure 2d), indicating that D112 inhibited mitochondrial respiration in the intact cells. Moreover, in the presence of the ATP synthase-inhibitor, oligomycin, D112-treated cells showed a relative increase in LEAK respiration, indicative of proton and electron leak across the inner membrane. To test whether D112 blocked electron flow, we dissipated the proton-motive force across the inner membrane with increasing concentrations of the uncoupler FCCP to induce maximal electron flow (ETS capacity). We found D112 decreased ETS capacity in a dose-dependent manner. Interestingly, 0.0625 *μ*g/ml D112 significantly decreased electron flow without increase in LEAK respiration, indicating that electron transport inhibition rather than mitochondrial membrane damage was the primary effect of D112 (Figure 2d). To localize the defect in ETS, we tested mitochondrial respiration in permeablized cells. We found routine respiration was decreased in 0.0625 *μ*g/ml D112-treated cells, whereas LEAK respiration was not affected (Figures 2e and f), confirming electron transport inhibition in the absence of mitochondrial membrane damage. Complex I respiration measured in the presence of malate and pyruvate (Figures 2e and f) or malate and glutamate (data not shown) and ADP was significantly blocked by D112, which indicated a defect in Complex I. In contrast, complexes II and IV respiration were unaffected (Figures 2e and f). Altogether, these studies demonstrated that D112 inhibited electron flow from Complex I of the ETS, identifying a mechanism for D112-mediated ROS production.

### ROS contributes to D112 selective cytotoxicity

**Figure 3:**
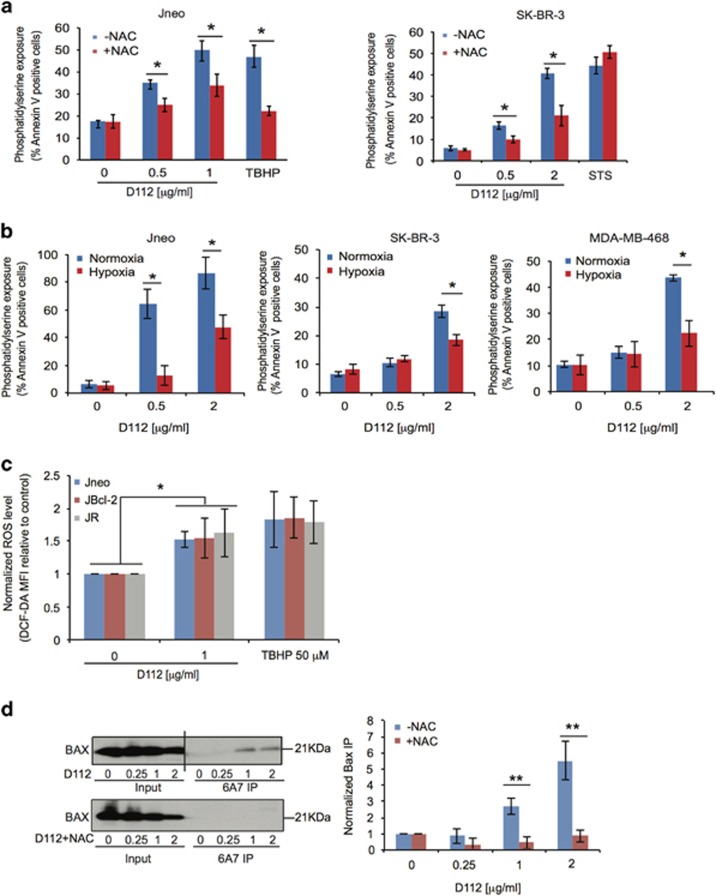
Functional involvement of ROS production in D112 toxicity. (**a**) D112-induced apoptosis in the presence or absence of NAC. Phosphatidylserine exposure was quantitated by flow cytometry. (**b**) D112-induced apoptosis in the presence or absence of oxygen. (**c**) D112-induced ROS production as determined by flow cytometry of CM-H_2_DCFDA. Normalized ROS level was MFI of (treated cells/untreated cells). (**d**) D112-induced Bax activation. Representative immunopreciptiation/western blots of activated Bax (6A7 IP). Bax activation was quantitated as band intensities of (immunoprecipated Bax/input Bax)/untreated (immunoprecipitated Bax/input Bax). All values are mean±S.D. of three independent experiments. *P<0.05, **P<0.01

To test functional relevance of D112-induced ROS, we pre-incubated cells with the ROS scavenger NAC (*N*-acetylcysteine) and observed that NAC significantly reduced D112-induced cell death (Figure 3a), while having no effect on the ROS-insensitive apoptosis inducer, staurosporine (STS). We confirmed the requirement of oxygen by measuring cell death under hypoxic conditions where ROS would be mitigated. Jneo, SK-BR-3 and MDA-MB-468 cells incubated in the absence of oxygen were all significantly resistant to D112-induced apoptosis (Figure 3b). Together, these observations indicate that ROS generation was necessary for D112-mediated cytotoxicity.

We next examined whether ROS production was an initiating event of D112-induced cell death. Previously, we showed that the pro-apoptotic protein BAX was required for D112-induced cell death,^1^ and it was inhibited by the anti-apoptotic oncogene Bcl-2.^1^ We examined ROS production in the Bcl-2 overexpressing cell line (JBcl-2)^1^ and BAX/BAK deficient cell line (JR).^1^ D112 induced a similar increase of ROS in all cell lines (Figure 3c), indicating that ROS production was upstream of Bcl-2 and Bax. Bax was activated in response to D112 as demonstrated by reactivity to the conformation-specific anti-Bax antibody 6A7^28^ in immunofluorescence (Supplementary Figure S5a). Moreover, active Bax was immunoprecipitated from D112-treated cells, whereas NAC ablated Bax activation, confirming that ROS was required for Bax activation (Figure 3d). Interestingly, D112-induced mtDNA damage was also partially protected by NAC (Supplementary Figures S5b and c). Altogether, these data demonstrate that D112-induced ROS production is essential for BAX activation and apoptosis.

**Figure 4:**
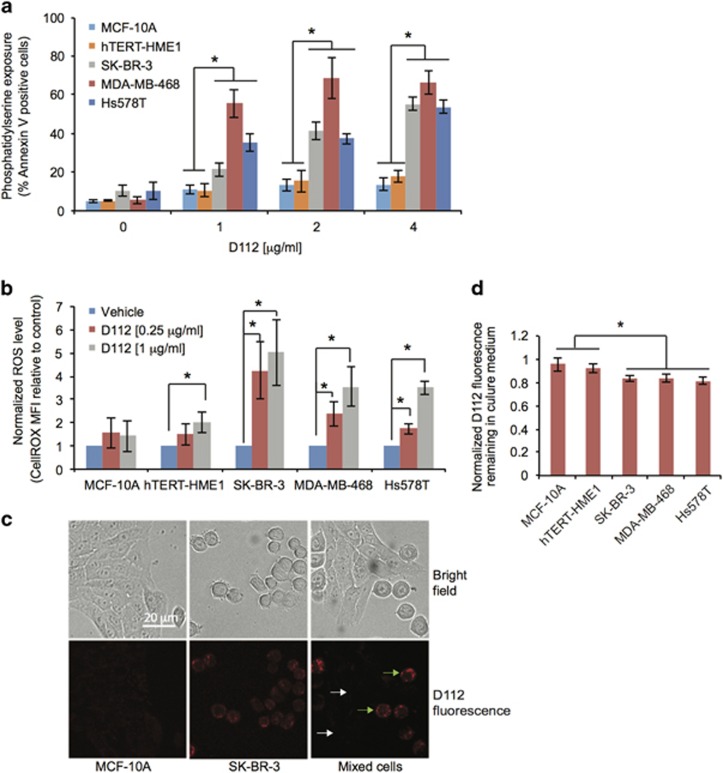
Contribution of ROS generation and preferential uptake in D112 selectivity. (**a**) D112 selectively induced apoptosis in cancer cells. Phosphatidylserine exposure was quantitated by flow cytometry. (**b**) D112 induced high level of ROS in cancer cells. Normalized ROS level was MFI of (treated cells/untreated cells). (**c**) Selective intracellular D112 uptake. White and green arrows indicate representative MCF-10A and SK-BR-3 cells. Scale bar, 20 *μ*m. (**d**) Quantitation of D112 uptake from media. Normalized D112 intensity was displayed as the ratio of D112 fluorescence in (media that contained cells/media without cells). All values are mean±S.D. of three independent experiments. *P<0.05

Cancer cells are highly sensitive to ROS-based therapies, due to an elevated oxidative stress environment.^29^ The requirement for ROS led us to investigate whether ROS production was the mechanism for D112-selectivity. We first verified cancer cell-selective toxicity of D112 in a panel of breast cell-derived cell lines (Figure 4a). Indeed, non-transformed MCF-10A and hTERT-HME1 cells were less sensitive to D112 than SK-BR-3, MDA-MB-468 and Hs578T carcinoma cell lines. We measured the base-line level of ROS in these cells and looked for an association of elevated ROS with D112-sensitivity. Surprisingly, MCF-10A and hTERT-HME1 showed higher basal ROS levels than SK-BR-3, MDA-MB-468 and Hs578T (Supplementary Figure S6a), inconsistent with a model whereby transformed cells harbor elevated ROS. Upon addition of D112, however, the cancer cell lines elevated ROS levels 3-4 fold, whereas the non-transformed cells showed limited ROS induction (Figure 4b). Together, these data support a model where D112 selective cancer cell killing is mediated by a burst in ROS levels.

### D112 is preferentially taken up by cancer cells

ROS induction in cancer cells may be a consequence of preferential D112 uptake, which would be in line with other DLCs that accumulate selectively in cancer cell mitochondria.^2, 30^ We had already shown that D112 enters cancer mitochondria^1^ (Supplementary Figure S6b). To directly compare D112 uptake, transformed SK-BR-3 and non-transformed MCF-10A cells were seeded in the same chamber and incubated with D112 (Figure 4c). We could distinguish cell lines through their distinctive morphology and observed that D112 accumulated to higher levels in SK-BR-3 cells (smaller round cells, see green arrows) as compared to MCF-10A cells (larger flat cells, see white arrows). Given that D112 fluorescence increases upon DNA binding, increased intracellular fluorescence could be due to increased uptake and/or binding of D112 to DNA. Thus, we also measured the fluorescence intensity remaining in the media after treatment (Figure 4d). There was significantly less fluorescence in the media of the cancer cell lines *versus* the non-transformed cell lines. Taken together, these results indicate that D112 accumulated preferentially in the carcinoma *versus* non-transformed cell lines.

Differential cellular uptake of other DLCs is facilitated by the elevated electrochemical potential (Δ*ψ*) of cancer mitochondria.^2, 6^ We first confirmed the requirement of mitochondrial membrane potential for D112 internalization. Treatment of SK-BR-3 cells with the mitochondrial uncoupling agent, carbonyl cyanide *m*-chlorophenyl hydrazine (CCCP), diminished intracellular fluorescence (Supplementary Figure S6c). Given that cancer cells are reported to have hyperpolarized mitochondrial membrane potential, this may explain why cancer cells take up more D112. However, using the electrochemical-sensing dye DiOC_6_ (3,3'-Dihexyloxacarbocyanine Iodide), we saw no correlation between cellular D112-sensitivity and Δ*ψ* (Supplementary Figure S6d). Thus, the mechanism of selective uptake remains unclear.

### Photo-activation of D112 increases its cytotoxic potential

**Figure 5:**
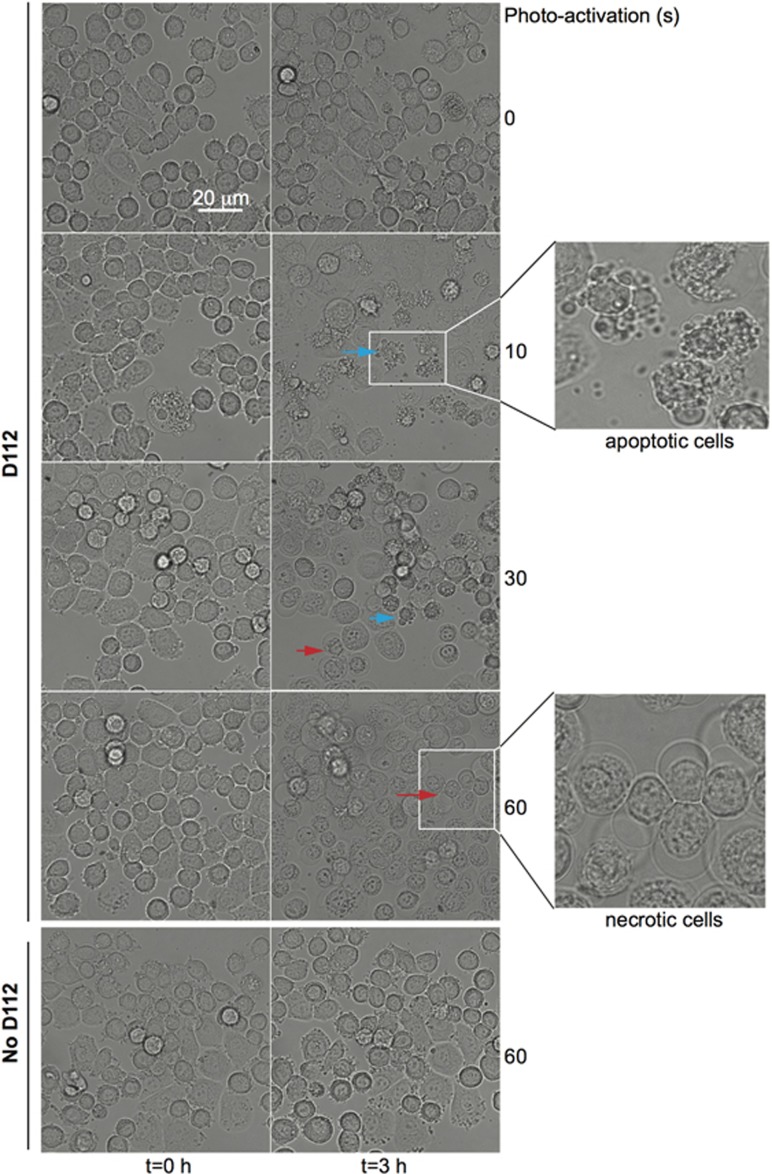
Effect of photo-activation on D112-induced cell death. SK-BR-3 cells were treated with 1 *μ*g/ml D112 for 1 h, washed, and photo-activated for the indicated times (10-60 s). Representative images are shown at 0 and 3 h incubation. Morphological cell death by apoptosis (blue arrow) or necrosis (red arrow) is indicated. Scale bar, 20 *μ*m

**Figure 6:**
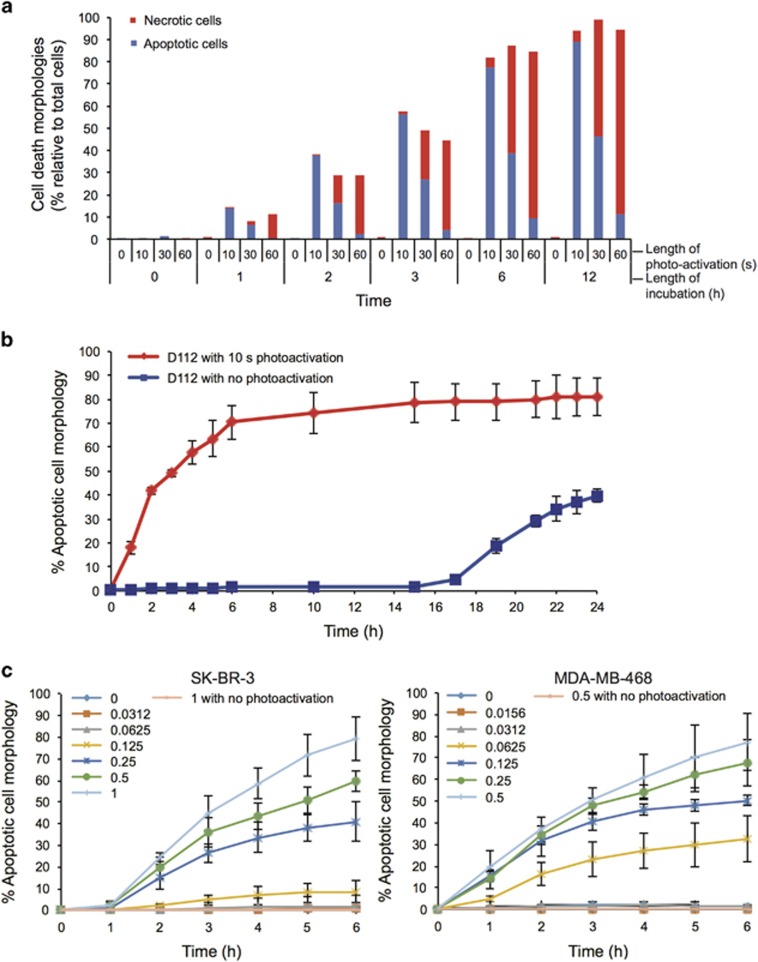
Characterization of photo-activation on D112-induced apoptosis. (**a**) Quantitation of cell death morphologies in SK-BR-3 cells. Cells were treated with 1 *μ*g/ml D112 for 1 h, washed, photo-activated for the indicated amounts of time (0-60 s) and cell death morphologies were quantitated at indicated times. (**b**) Kinetics of apoptosis-induction by photo-activated D112 in SK-BR-3 cells. Cells were treated with 1 *μ*g/ml D112 for 1 h, photo-activated for 10 s, and percent apoptotic cells were recorded for the indicated time points. (**c**) Dose-dependent analysis of photo-activated D112-induced apoptosis in SK-BR-3 and MDA-MB-468 cells. Cell lines were treated with D112 at the indicated concentrations for 1 h, washed, photo-activated for 10 s, and percent apoptotic cells were quantified at the indicated timepoint. Each data point is an average of three random fields of view per experiment taken from three independent experiments. All values are mean±S.D. of three independent experiments

Kodak Laboratories originally developed D112 for use as a photosensitizer in photographic emulsions. Photosensitizers produce ROS by transferring light energy to oxygen.^31^ An exciting application of photosensitizers is their use in PDT that combines low-dose drug treatment with targeted activation via light therapy.^32^ A photosensitizer is a light-absorbing compound that is activated upon exposure to specific wavelengths of light. To return to the ground state, the photosensitizer transfers energy or charge to cellular substrates, such as lipid membranes or DNA, or to oxygen to generate ROS.^31^ We therefore examined whether light activation increased D112 efficacy. To mimic a PDT-application, SK-BR-3 cells were incubated for 1 h, rinsed with fresh medium, and then exposed to a single pulse (0, 10, 30 or 60 s) of 541 nm laser light and cell morphology was recorded 3 h later (Figure 5). We observed various cell death morphologies with differing lengths of light exposure. Specifically, a 10 s treatment induced apoptotic morphology (shrunken and blebbing cells with fragmented bodies, see blue arrows), while a longer photo-activation pretreatment (60 s) induced necrotic morphology (swollen cell body with vacuoles, see red arrows). An intermediate pulse (30 s) induced mixed apoptotic and necrotic cells (Figure 6a). Importantly, there was no D112-induced cell death in the absence of photo-activation under these conditions, nor did light pretreatment without D112 cause cell death (Figure 5). Comparing the dynamics, photo-activated cells reached half-maximal cell death by 2 h, whereas non-photo-activated cells required 24 h to reach similar levels of cell death (Figure 6b). Finally, dose response curves demonstrated that photo-activated D112 induced significant cell death at a concentration as low as 0.25 and 0.0625 *μ*g/ml in SK-BR-3 and MDA-MB-468 cells, respectively, whereas no cell death was observed at up to 1 *μ*g/ml of non-activated D112 (Figure 6c). Clearly photo-activation greatly increased D112 activity as a cytotoxic agent.

**Figure 7:**
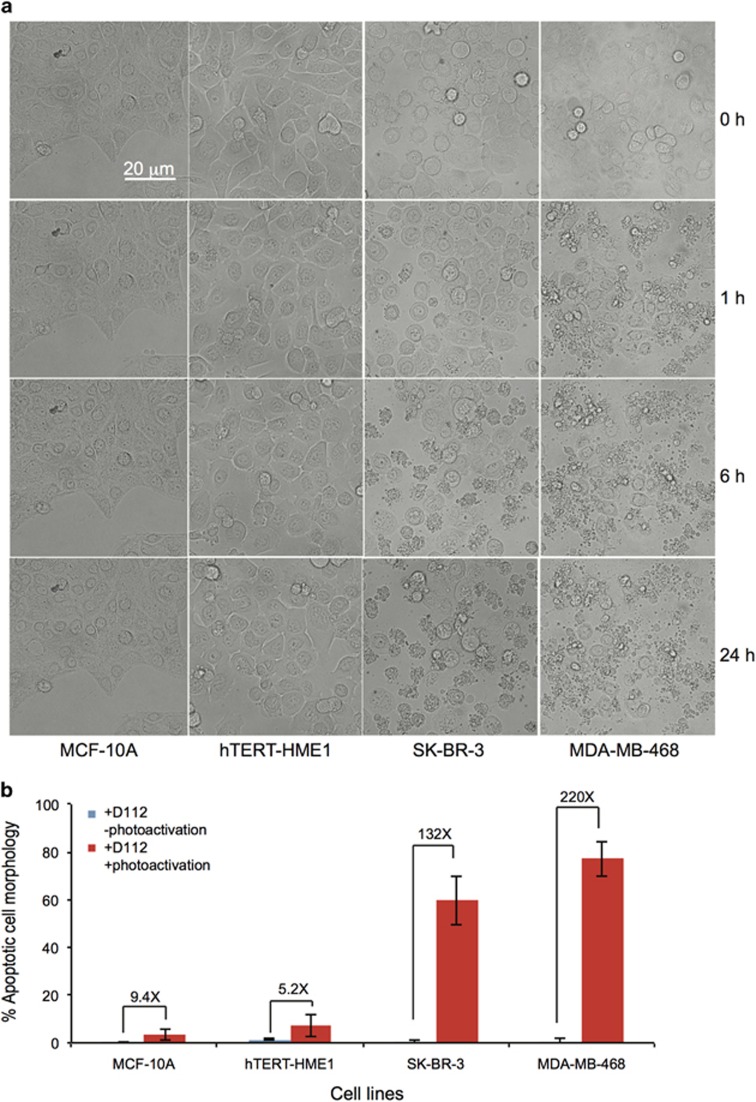
Comparison of photo-activated D112-induced cell death in transformed and non-transformed cell lines. (**a**) Cell lines were pre-treated with 0.5 *μ*g/ml D112 for 1 h, photo-activated for 10 s and apoptotic morphology was recorded at indicated time points (0-24 h). (**b**) Percent of apoptotic cells was quantified for the indicated cell lines in the presence or absence of photo-activation at the 6 h timepoint. Fold induction of apoptosis in response to photo-activation is indicated. All values are mean±S.D. of three independent experiments

**Figure 8:**
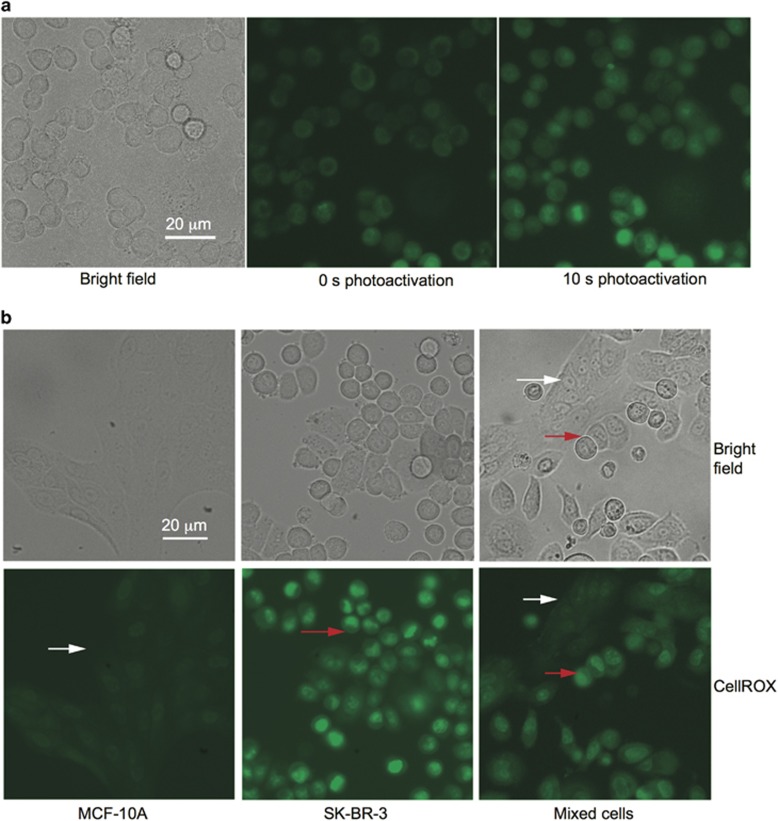
Analysis of ROS production in response to D112-photo-activation. (**a**) CellROX green fluorescence of 0.5 *μ*g/ml D112-treated SK-BR-3 cells with and without photo-activation. (**b**) CellROX green fluorescence of 10 s photo-activated D112-treated MCF-10A and SK-BR-3. White and red arrows indicate representative MCF-10A and SK-BR-3 cells, respectively. Scale bar, 20 *μ*m.

To determine whether the photo-activation of D112 retained selectivity to cancer cell lines, we quantified D112-induced apoptosis in non-transformed cell lines and cancer cell lines. Photo-activated D112 increased cell death 132-fold for SK-BR-3 and 220-fold for MDA-MB-468 cancer cell lines and 9- and 5-fold for non-transformed MCF-10A and hTERT-HME1 cells (Figures 7a and b). We also observed elevated ROS upon photo-activation, indicating that enhanced cell death was mediated by PDT-based ROS production (Figure 8a). We further compared the intracellular level of ROS between the two cell types under PDT conditions (Figure 8b). SK-BR-3 cells (see red arrows) showed greater intensity of CellROX fluorescence than MCF-10A cells (see white arrows) in response to photo-activation. Thus, D112 selective toxicity was associated with ROS production in PDT. All together, the data indicate that photo-activation of D112 greatly increases its cytotoxicity and potentiates the selectivity of D112 against cancer cells.

## Discussion

Based on the data presented here, we propose a model for D112-induced cytotoxicity. D112 enters cells and localizes to mitochondria in a Δ*ψ*-dependent manner. Once in the mitochondria, D112 binds mtDNA and inhibits electron flow through the ETS, leading to ROS production, mtDNA damage, activation of Bax and induction of apoptosis. Cancer cells take up D112 more efficiently than non-transformed cell lines. Photo-activation potentiates the ability of D112 to produce ROS either by direct transfer of electrons to oxygen, or via ETS-mediated ROS generation. The combined effect of selective uptake and photo-activation-enhanced ROS production leads to cancer-cell selective apoptosis by D112.

Given the role of mitochondria in the generation of metabolic energy and regulation of apoptosis, DLCs, such as D112, are of interest as cancer therapeutics. DLCs block mitochondrial metabolism through distinct mechanisms.^2^ The DLC compound, dequalinium chloride, binds to the ubiquinone and inhibitor binding pocket of complex I,^33^ whereas MKT-077 inhibition of electron transfer is due to a more generalized disruption of ETS components.^34^ We demonstrated that D112 also interrupted electron transfer at Complex I. Inhibition of electron flow produces ROS^35^ due to leak of electrons from the electron transport channel and reaction with oxygen to produce superoxide ROS. D112-mediated block of electron flow led to increased ROS levels. Downregulation of ROS by growth in hypoxic conditions and rho^0^ cells decreased D112 sensitivity, whereas stimulation of respiration by growth in YPG sensitized cells to D112 toxicity. All together these results indicated that D112-induced ROS production was linked to Complex I inhibition and rationally suggest that combining D112 with glycolytic inhibitors such as 2-deoxyglucose^36^ could further sensitize tumor cells to D112-induced apoptosis.

The resistance of rho^0^ cells also suggested the involvement of mtDNA in D112 cytotoxicity. Unlike the nuclear genome, mtDNA is more susceptible to ROS-induced damage.^22, 37^ Several DLC agents, such as DECA^38^ and MKT-077^37^ induce mtDNA damage in cells. We identified that D112 could bind to DNA, induce mtDNA strand breaks and generate petite strains in yeast. The ROS scavenger NAC partially rescued D112-mediated DNA damage, suggesting that a ‘vicious cycle' occurred in D112-treated cells. Potentially, the production of ROS-induced mtDNA damage initially leads to progressive respiratory system dysfunction and further increases ROS production.

D112 preferentially induced ROS levels in transformed cells. Moderate ROS levels increase cell proliferation and differentiation, whereas excessive levels of ROS cause oxidative damage to lipids, proteins and DNA leading to cell death.^39^ During mitochondrial respiration, a leak of electrons to oxygen, mostly from complex I and complex III, generates superoxide making mitochondria the major source of ROS.^40^ Inhibitors that block electron transport elevate ROS^41^ and we showed that D112-induction of ROS was due to inhibition of Complex I respiration. In support of this another DLC (FPB) that blocks ETS, also induces ROS.^42^ Compared to normal cells, cancer cells have increased levels of ROS due to their rapid proliferation rate,^25, 27^ although we did not observe this trend in the cell lines in our study. Nevertheless cancer cells are documented to have a diminished capacity to deal with ROS, which sensitizes them to exogenous ROS insults.^26, 29^ In line with this, we observed that D112 treatment elevated ROS levels to a greater extent in transformed cells. This suggests that transformed cells may be limited in their capacity to buffer against bursts in ROS making them sensitive to ROS-inducers, such as D112.

We had previously shown that Bax was critical for D112-induced apoptosis.^1^ In response to apoptotic stimuli, Bax undergoes conformational changes, oligomerization and insertion into the mitochondrial outer membrane to create pores that release death factors from the mitochondria.^43^ Here we show that D112-induced Bax activation was dependent on ROS. Previous studies have demonstrated that ROS or perturbation of the intracellular redox causes Bax translocation and oligomerization via distinct pathways.^44, 45^ Further investigation is needed to elucidate the mechanism(s) specifically involved in D112-induced Bax activation by ROS.

Finally, we find that D112-induced ROS production and cell killing was potentiated by photo-activation, indicating that D112 may have applications in PDT. PDT spares normal tissue by delivering non-toxic doses of a photosensitizer that is subsequently light-activated at targeted tumor sites.^46^ To date, several PDT drugs have been approved for oncological applications.^46^ Limitations of PDT include skin sensitivity and light source challenges, and efforts are underway to identify new photosensitizers with more selective uptake by tumor cells and stronger absorption at longer wavelengths. Of particular interest are photosensitizers that localize to mitochondria promoting apoptosis, since photosensitizers targeting the plasma membrane or lysosomes may block the apoptotic program.^47^ Thus, photo-activatable DLCs may be ideally suited for PDT. In particular, photo-activation of MKT-077 enhanced its ability to damage the electron-transport of mitochondria *in vitro*,^48^ and photo-activation of Rh-123 inhibited xenograft tumor growth in mice.^49^ To our knowledge, our report is the first to demonstrate that photo-activation potentiates cancer cell-selective killing. This selectivity is achieved through a combination of selective D112 uptake that inhibits electron transport through Complex I to generate ROS, which is less tolerated by cancer cells. Photo-activation amplifies this ROS-generation step to ultimately trigger Bax-dependent apoptotic pathways. Thus D112 may be a worthwhile candidate for further evaluation in a PDT setting.

## Materials and Methods

### Cell culture and chemicals

Jurkat cell lines Jneo and JBcl-2 were kindly provided by Dr. Chris Bleackley (University of Alberta), JR cells were kindly provided by Dr. Hannah Rabinowich (University of Pittsburgh). The human normal breast epithelial cell lines MCF-10A and hTERT-HME1 and the breast carcinoma cell line Hs578T were obtained from ATCC (Manassas, VA, USA). All yeast strains were obtained from the MAT *α* yeast deletion collection.^50^ zVAD-fmk was purchased from BD Pharmingen (Mississauga, ON, Canada); CellRox Green (C10444), CM-H_2_DCFDA (C6827) and Alexa Fluor 647 Annexin V conjugate (A23204) were obtained from Invitrogen (Carlsbad, CA, USA). All chemicals were purchased from Sigma-Aldrich (St Louis, MO, USA) unless indicated otherwise.

### D112 localization in yeast

3 × 10^6^ yeast cells were treated with 5 *μ*g/ml D112 for indicated times, washed in fresh YPD medium, and imaged using a Zeiss AxioObserver Z1 Microscope with × 40 objective lens. D112 fluorescence was detected using the Chroma Filter Set 49005 (Cy3, excitation 541/30, emission 620/60).

### Yeast growth and viability assays

Yeast doubling times were determined from the maximum slope of exponential growth curves. For viability assays, after D112 treatment (5 *μ*g/ml in YPD and 0.625 *μ*g/ml in YPG), equivalent yeast cells were washed and serially diluted in fresh YPD medium and spotted on YPD plates. For quantification of viability, equivalent yeast cells were incubated on YPD plates and colony number was counted.

### D112 spectral analysis

Fluorescent spectral scans were performed using a model 814 photomultiplier detection system (Photon Technology International, London, ON, Canada) and PTI FeliX32 software. D112 (0.25 *μ*g/ml), in the presence or absence of DNA, was excited from 340 to 620 nm with an interval wavelength of 20 nm. For each excitation wavelength, the corresponding emission spectra were collected from the excitation wavelength +20 to 750 nm. To detect D112 cellular uptake by measuring residual D112 in the media, medium was collected from cells that had been treated with D112 for 1 h. D112 fluorescence emission was normalized to control medium without cells.

### ROS detection

For yeast cells, 5 *μ*M CellROX green was added to the medium, incubated at 30°C for 20 min. CellROX Green was detected using Chroma filter set 49002 (GFP, excitation 470/40 nm, emission 525/50 nm). For mammalian cells, 2.5 *μ*M CellRox Green reagent was added to the cells and incubated for 30 min at 37°C. ROS levels were detected using a BD Accuri C6 flow cytometer and mean fluorescence intensity (MFI) was quantified in the FL-1 channel. ROS production was also examined using CM-H_2_DCFDA. 1.5 *μ*M CM-H_2_DCFDA was incubated with cells for 15 min, and CM-H_2_DCFDA fluorescence was detected by flow cytometry. H_2_O_2_ (400 *μ*M) and TBHP *(tert*-butyl hydroperoxide, 200 *μ*M) were used as positive controls. For ROS scavenger experiments, cells were pre-treated with NAC (10 mM) for 1 h.

### High-resolution respirometry

1~2 × 10^6^/ml cells were treated with D112 at indicated concentrations for 30 min, rinsed with RPMI and re-suspended in fresh medium to a concentration of 1.5 × 10^6^ per ml. Two ml of cells were loaded into each of four respirometry chambers (Oxygraph-2k, Oroboros Instruments Inc., Innsbruck, Austria), and oxygen consumption was measured as a function of time. First, the cellular respiration was measured in the presence of endogenous substrates (Routine). Oligomycin (Omi; 2 *μ*g/ml) was then added to inhibit ATP synthase allowing measurement of LEAK oxygen flux, compensating for proton leak, proton slip, cation cycling and electron leak.^51^ The stepwise addition of the uncoupler FCCP (stepwise additions of 0.125 *μ*M) was performed to determine the maximal electron transport capacity (ETS). Rotenone (Rot; 0.5 *μ*M) and Antimycin A (Ama; 2.5 *μ*M) were then added to inhibit Complexes I and III.

To test respiration in permeabilized cells, cells were re-suspended in MiR05 medium after treatment. After measurent of Routine respiration in the presence of endogenous substrates, Complex I substrates malate and pyruvate (M and P; 2 and 5 mM, respectively) were added and the LEAK state was initiated by permeabilization of the plasma membrane with addition of digitonin (Dig; 3.75 *μ*g/ml; concentration determined with separate experiments including a digitonin titration in the presence of succinate, rotenone and ADP). Coupled OXPHOS (Oxidative phosphorylation) for Complex I respiration (CI) was then measured after addition of saturating ADP (2.5 mM). Cytochrome *c* (Cyt; 10 *μ*M) was added to test the integrity of outer mitochondrial membrane. The following succinate (S; 10 mM) addition allowed the measurement of respiration with convergent electron flow through Complexes I and II (CI&II). Rotenone (0.5 *μ*M) was then added to inhibit Complex I allowing measurement of Complex II respiration (CII). Addition of antimycin A (2.5 *μ*M) completely inhibited electron transport. Complex IV respiration was measured after addition of Ascorbate (Asc; 2 mM) and tetramethyl-phenylenediamine (TMPD; 0.5 mM), followed by addition of inhibitor sodium azide (Azd; 100 mM).

### Immunoprecipitation

Cells were lysed in CHAPS lysis buffer (1% CHAPS; 150 mM NaCl; 50 mM Tris pH7.4; 2 mM EDTA; protease/phosphatase inhibitors), and cleared supernatant was incubated with Bax 6A7 antibody (B8429, Sigma-Aldrich, St Louis, MO, USA) followed by immunoprecipitation using protein A-coupled Sepharose beads. Immunoprecipitated products were separated by SDS-PAGE followed by western blotting with primary antibody against Bax (2772, Cell Signaling, Boston, MA, USA).

### Hypoxia treatment

Hypoxia treatment was performed in a hypoxia chamber using a Xvivo closed incubation system, with 0% oxygen present. Cells were placed in the chamber 4 h before D112 addition for 24 h.

### Determination of apoptosis

Apoptosis was determined by analyzing phosphatidylserine exposure using Alexa Fluor 647 Annexin V conjugate staining as described previously.^1^

### D112 photo-activation and ROS measurement

Cells were incubated with D112 at indicated concentrations for 1 h in the dark and then rinsed twice with fresh medium. Cells were subjected to photo-activation (excitation 541/30 nm) and cell images were taken under microscope with × 20 objective lens at the indicated times.

### Statistical analysis

Statistical significance was determined using a two-tailed Student's *t*-test for two means with equal variance. For statistical analysis of multiple groups (Figures 2 and 3), the one-way Analysis of Variance (ANOVA) test was performed and *P*-values were obtained by Tukey's *Post Hoc* test.
