# Supplementary material for: Photo-activation of the delocalized lipophilic cation D112 potentiates cancer selective ROS production and apoptosis
Source: Cell Death Dis. 2017 Feb 2;8(2):e2587–. doi: 10.1038/cddis.2017.19 (PMC5386467; doi:10.1038/cddis.2017.19)
Supplement: Supplementary Information [file cddis201719x1.pdf]

Supplementary figure legends

Supplementary Table 1 Yeast mutant strains. All strains were obtained from the MAT α yeast deletion collection.

Supplementary Figure 1 D112 is cytotoxic to yeast cells. (**a**) Yeast growth curve in YPD following treatment with D112 at the concentrations (μg/ml) shown. (**b**) Yeast viability following D112 treatment in YPD. After incubation with 5 μg/mL D112 for 24 h, yeast cells were plated to quantify viability in a colony formation assay. (**c**) Yeast growth curve in YPG following treatment with D112 at the concentrations (μg/ml) shown. (**d**) Yeast viability following D112 treatment in YPG. After incubation with 0.625 μg/ml D112 for 24 h, yeast cells were plated on YPD plates to quantify viability in a colony formation assay. All values represent the mean ± SD of three independent experiments. *$p<0.05$, **$p<0.01$, ***$p<0.001$.

Supplementary Figure 2 Mitochondrial respiration is not required for D112-induced proliferative delay. (**a**) Doubling times of indicated yeast cell strains grown in YPD. (**b**) Yeast mutant viability following D112 treatment and recovery on YPD. All values represent the mean ± SD of three independent experiments. *$p<0.05$, **$p<0.01$, ***$p<0.001$.

Supplementary Figure 3 D112 binds to nucleic acid *in vitro* and induces petite phenotype in yeast. (**a**) D112 electrophoretic assay displaying D112 fluorescence (left) or DNA fluorescence (right). (**b**) Induction of petite phenotype by D112-treatment of the W303-1A yeast strain as indicated by loss of red-pigmented

colonies. Ethidium bromide was used as a positive control. (**c**) Agarose gel of PCR-generated large and small mitochondrial DNA fragments from Jneo cells pre-treated as indicated. (**d**) Relative intensity was reported as intensity of (large fragment/small fragment of D112-treated cells)/ (large fragment/small fragment of non-D112-treated cells).

Supplementary Figure 4 D112 induces ROS production as measured by the ROS indicator CM-H_2DCF-DA. (**a**) Jneo and SK-BR-3 cells were treated with D112 or TBHP at indicated concentrations. CM-H_2DCF-DA fluorescence was measured by flow cytometry. (**b**) Normalized DCF-DA fluorescence intensity in indicated cell lines is displayed as mean fluorescence intensity of (treated cells/untreated cells). TBHP was used as positive control. All values are the mean ± SD of three independent experiments. *$p<0.05$, **$p<0.01$, ***$p<0.001$.

Supplementary Figure 5 Bax activation and the contribution of ROS to mtDNA damage. (**a**) Active Bax in D112-treated cells in immunofluorescence assay. (**b**) Agarose gel of PCR-generated large and small mitochondrial DNA fragments from Jneo cells pre-treated as indicated. (**c**) Relative intensity was reported as intensity of (large fragment/small fragment of D112-treated cells)/ (large fragment/small fragment of non-D112-treated cells).

Supplementary Figure 6 Basal ROS levels and mitochondrial potential in non-transformed and transformed cell lines. (**a**) CellRox Green fluorescence of indicated cell lines were quantitated by flow cytometry. (**b**) Confocal mitochondrial localization of D112 with linescan analysis in MDA-MB-468 and

Hs578T breast cancer cell lines. (**c**) D112-uptake in SK-BR-3 cells pre-treated with carbonyl cyanide m-chlorophenylhydrazone (CCCP, 100 μM). (**d**). Mitochondrial membrane potential in indicated cell lines as assessed by DiOC6 fluorescence and measured by flow cytometry. Mean ± SD of three independent experiments performed in triplicate are shown. *$p<0.05$.

Supplementary Methods

D112 electrophoretic gel-shift assay

0.025 mg/ml D112 was mixed with 0.5 μg or 1 μg DNA ladder and incubated for 30 min at room temperature in the dark. The mixture was loaded onto a 1% agarose gel and run at 100 V for 1 h. The gel was first exposed at 550/570 nm to capture the D112 fluorescent signal. The same gel was then stained with SYBR safe (S33102, Sigma Aldrich) to visualize the DNA at 492/510 nm.

D112 localization

Experiments were performed as described before [1].

Induction of the petite phenotype

An overnight culture of the W303-1A yeast strain was subcultured into fresh YPD at OD600 0.1. Two or 5 μg/ml D112 was added to the cell culture and incubated at 30°C with orbital shaking at 200 rpm for 4 h. Cells were diluted and counted under microscopy. ~300 cells were plated on YPD plates and incubated for 3 days at 30°C. The number of red and white colonies was manually counted. Ethidium bromide (EB, 20 μM) was used as a positive control.

mtDNA damage assay

Jneo cells were treated with 0.25 or 2 μg/ml D112 for 1 h. Genomic DNA was extracted using the Mammalian Genomic DNA Miniprep Kit (G1N70, Sigma Aldrich), according to the manufacturer's instructions. PCR was performed using Elongase Enzyme kit (10480028, ThermoFisher Scientific). Reactions were performed on the Bio-Rad C1000 Touch Thermal Cycler. The primers for large mtDNA fragment (8.9 kb) were 5'-TTTCATCATGCGGAGATGTTGGATGG-3' and 5'-TCTAAGCCTCCTTATTCGAGCCGA-3'; the primers for small mtDNA fragment (212 bp) were 5'-TTTCATCATGCGGAGATGTTGGATGG-3'and 5'-CCCCACAAACCCCATTACTAAACCCA-3'. The PCR program for the large mtDNA fragment was: cycle 1(1X), 94°C for 30 sec; cycle 2 (20X), step 1 at 94.0°C for 30 sec; step 2 at 64.0°C for 30 sec; step 3 at 68°C for 9 min; cycle 3 (1X). The PCR program for the small mtDNA fragment: cycle 1(1X), 94°C for 30 sec; cycle 2 (20X), step 1 at 94.0°C for 30 sec; step 2 at 64.0°C for 30 sec; step 3 at 72°C for 30 sec; cycle 3 (1X). Equal volumes of the PCR reactions were electrophoresed on an agarose gel and visualized by SYBR safe staining. A 15% agarose gel was used for the small mtDNA fragment detection, whereas an 8% agarose gel was used for detection of the large mtDNA fragment.

Immunofluorescence assay

SK-BR-3 cells were seeded on coverslips and treated with 1 μg/ml D112 for 16 h. Cells were then fixed with 4% formaldehyde solution and permeabilized with PBS containing 0.2% (v/v) Triton X-100. After blocking with 1% BSA, cells were

sequentially incubated with primary antibodies Bax 6A7 (B8429, Sigma) and Tom 20 (sc-11415, Santa Cruz) at 4˚C overnight, and then secondary antibodies, Alexa Fluor 488 (A-11001, ThermoFisher) and Alexa Fluor 594 (A-11037, ThermoFisher) at room temperature for 1 h. Coverslips were mounted with Prolong Gold (P36930, ThermoFisher) overnight and images were then taken on a Zeiss LSM 710 inverted confocal microscope fitted with a 40x 1.4 NA Oil DIC Plan-Apochromat objective (Carl Zeiss, Canada).
