# Supplementary figures and images for: Photo-activation of the delocalized lipophilic cation D112 potentiates cancer selective ROS production and apoptosis
Source: Cell Death Dis. 2017 Feb 2;8(2):e2587–. doi: 10.1038/cddis.2017.19 (PMC5386467; doi:10.1038/cddis.2017.19)

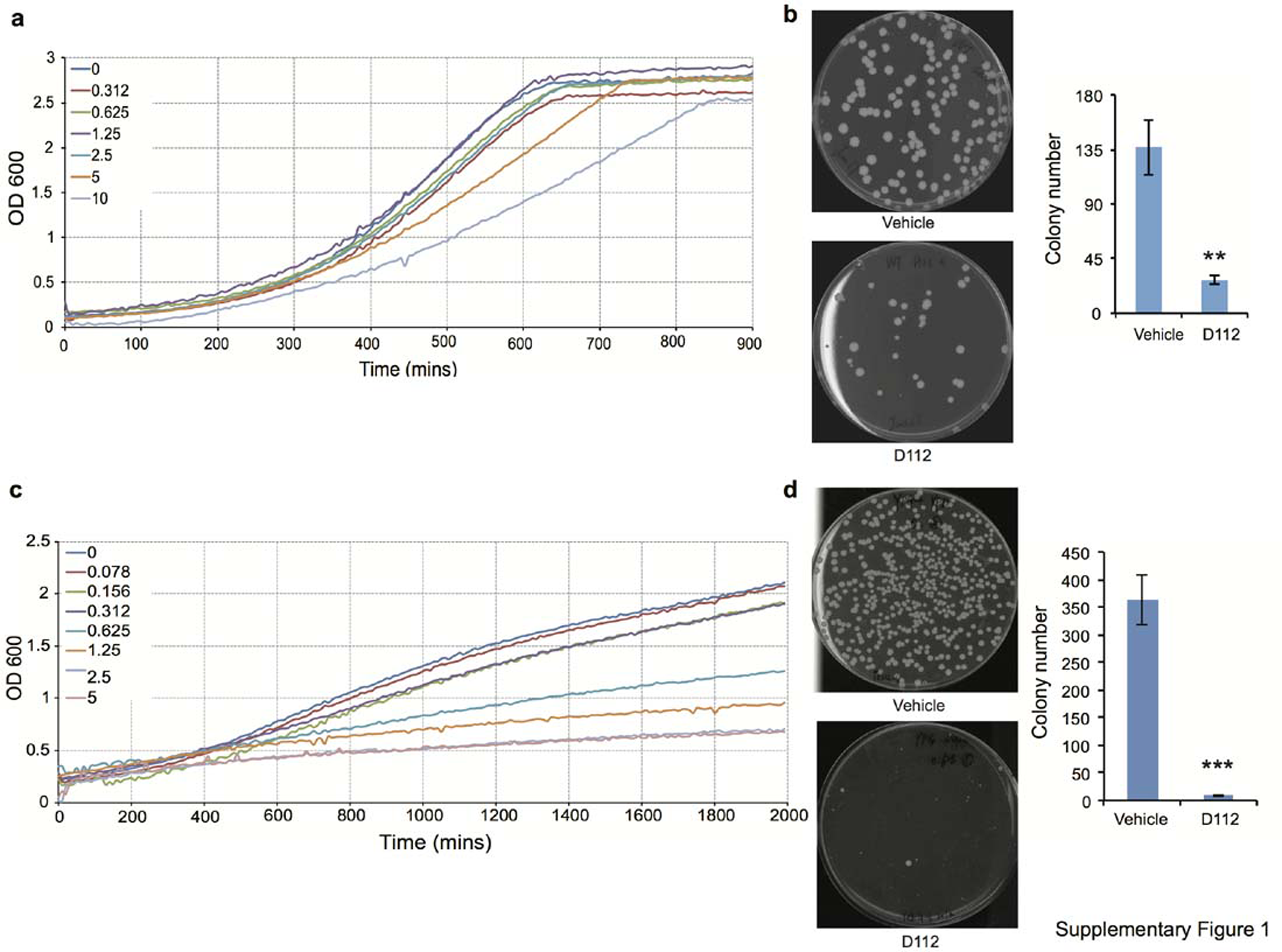

Supplement: Supplementary Figure 1 [file cddis201719x2.tif]

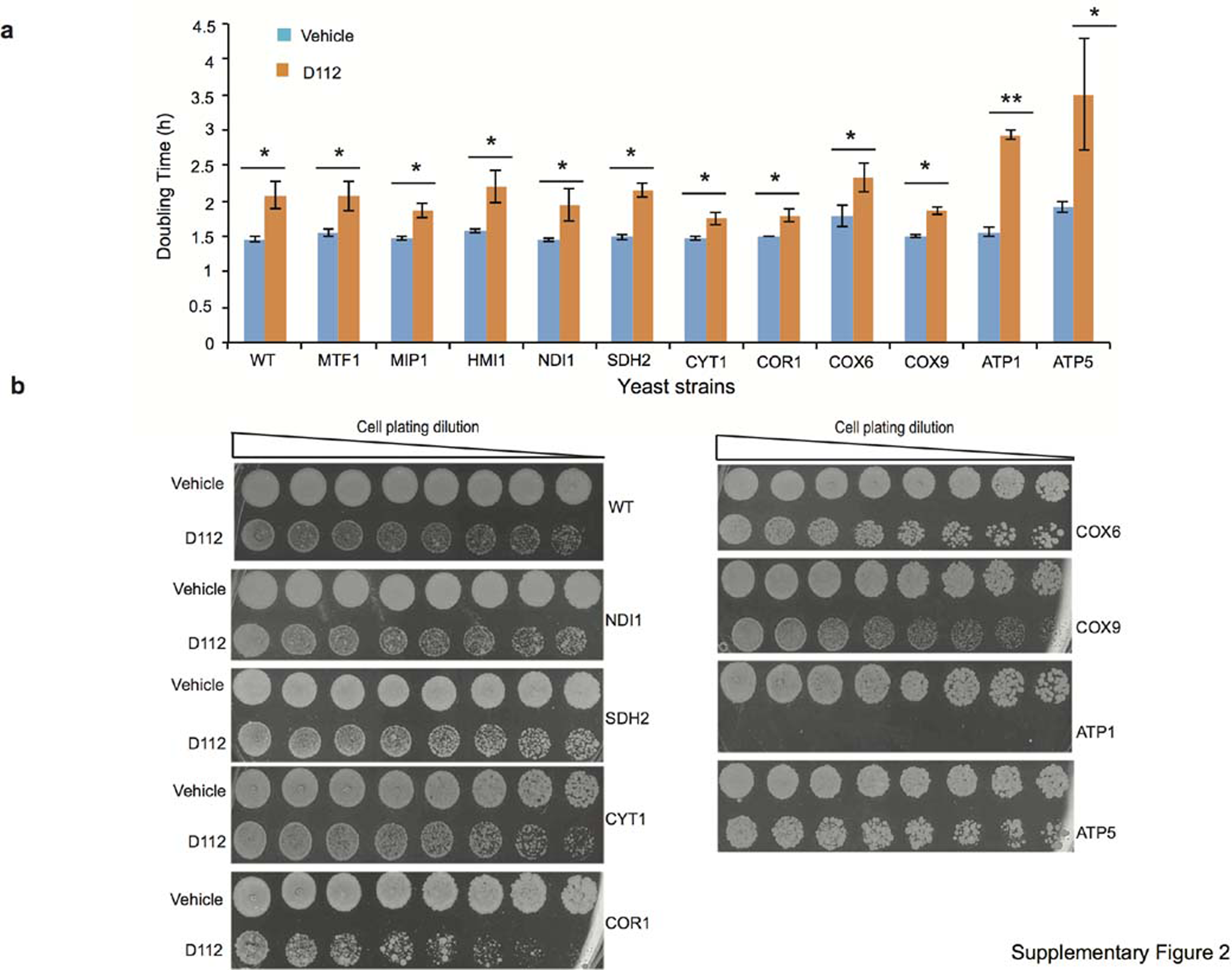

Supplement: Supplementary Figure 2 [file cddis201719x3.tif]

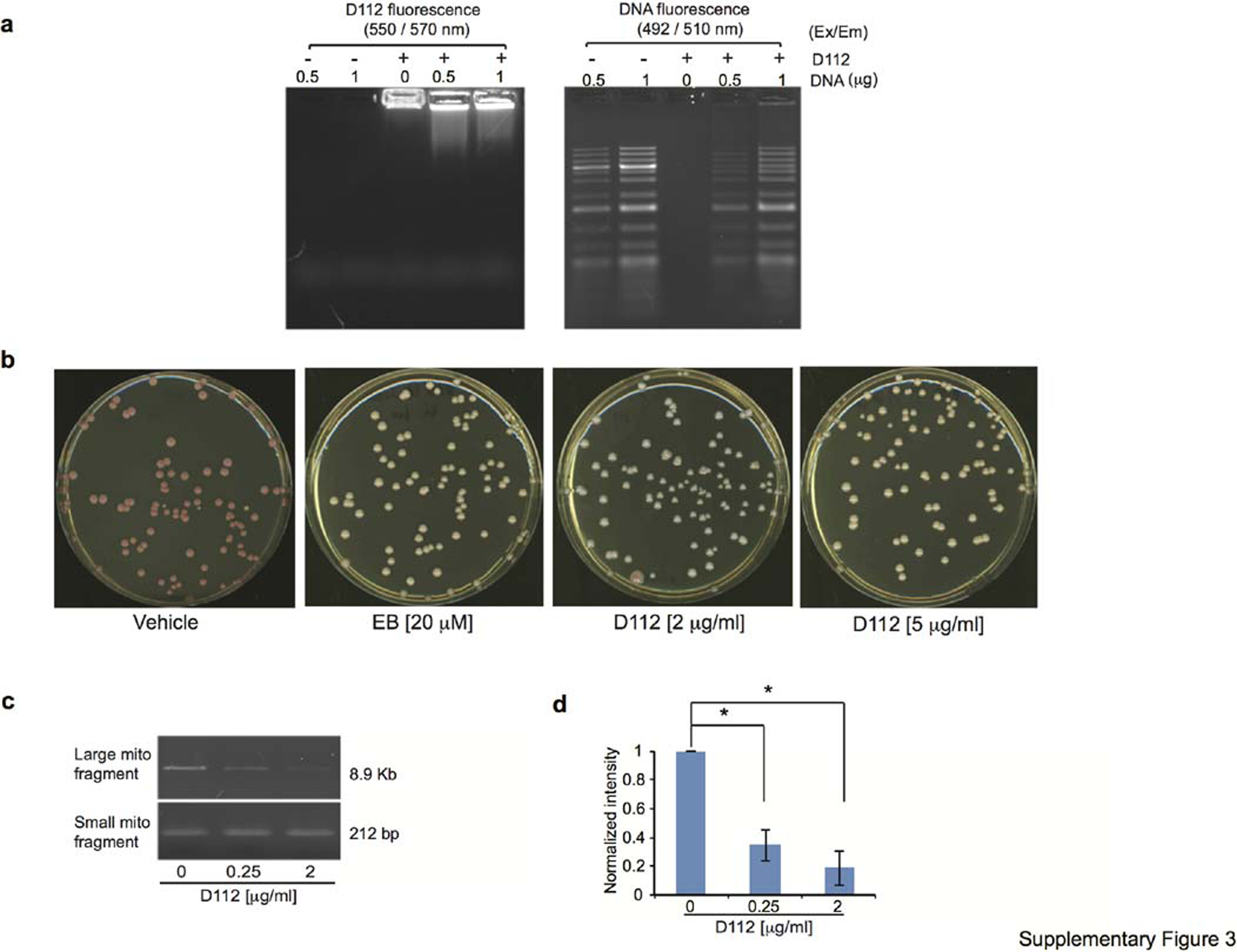

Supplement: Supplementary Figure 3 [file cddis201719x4.tif]

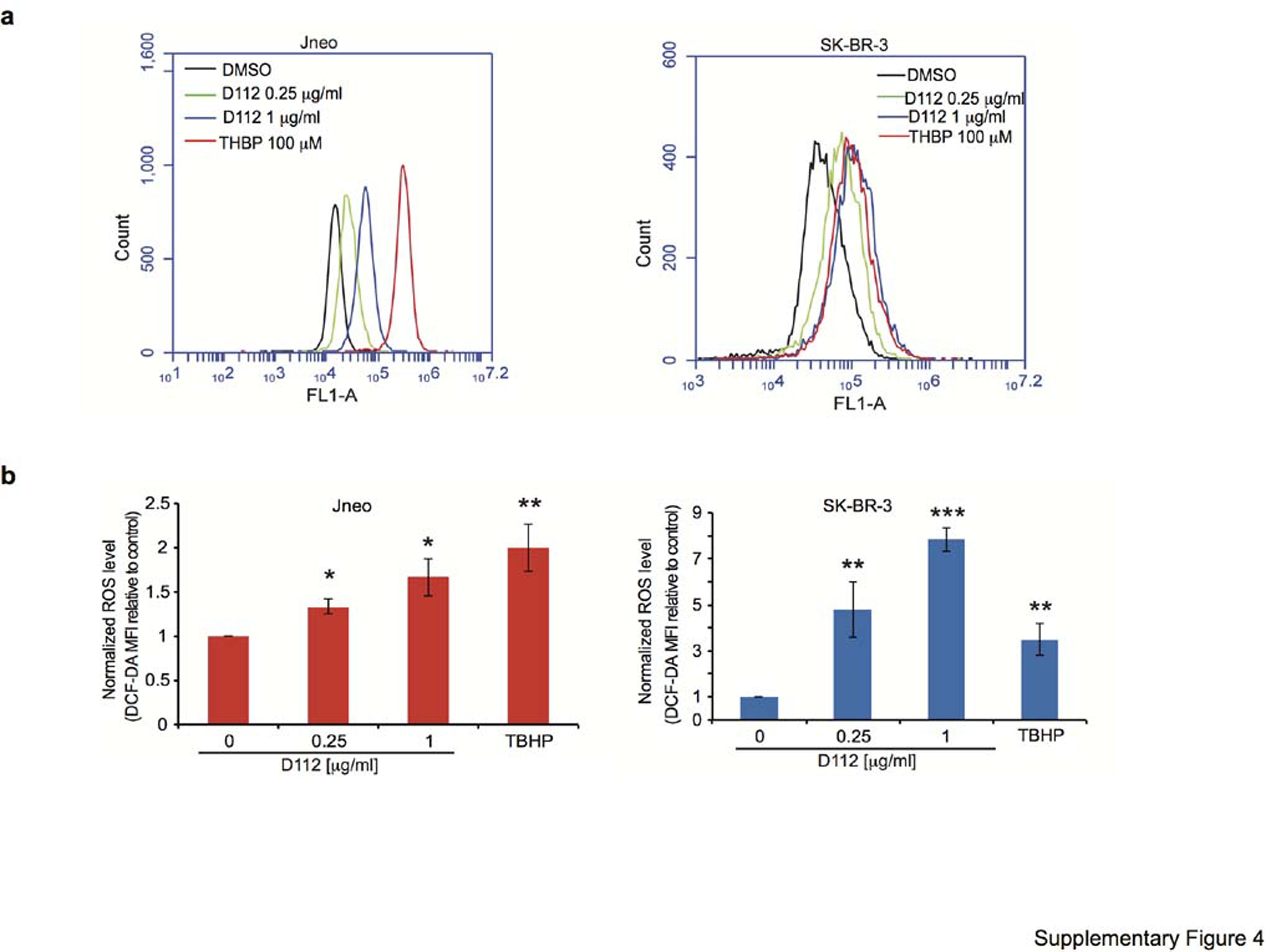

Supplement: Supplementary Figure 4 [file cddis201719x5.tif]

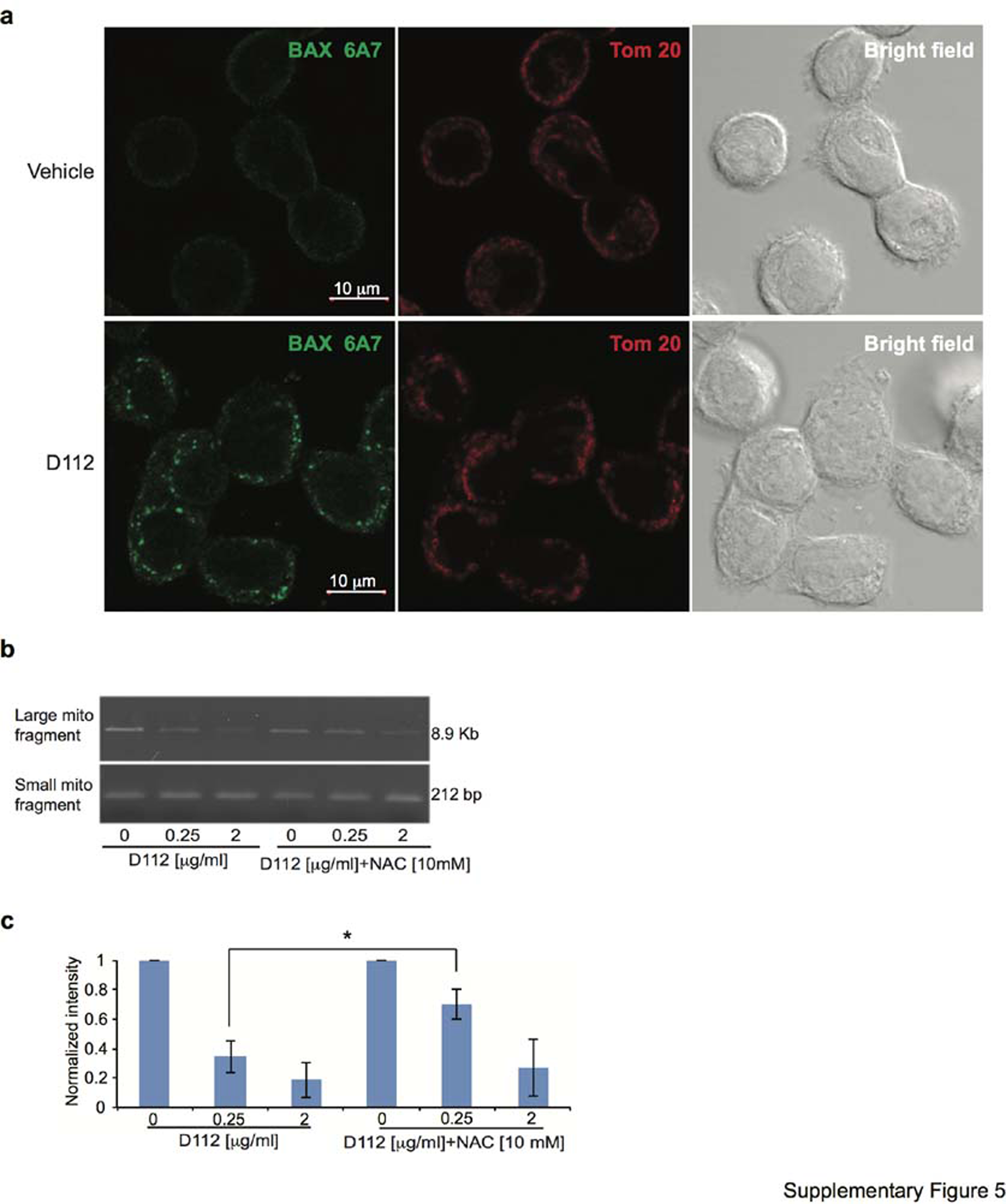

Supplement: Supplementary Figure 5 [file cddis201719x6.tif]

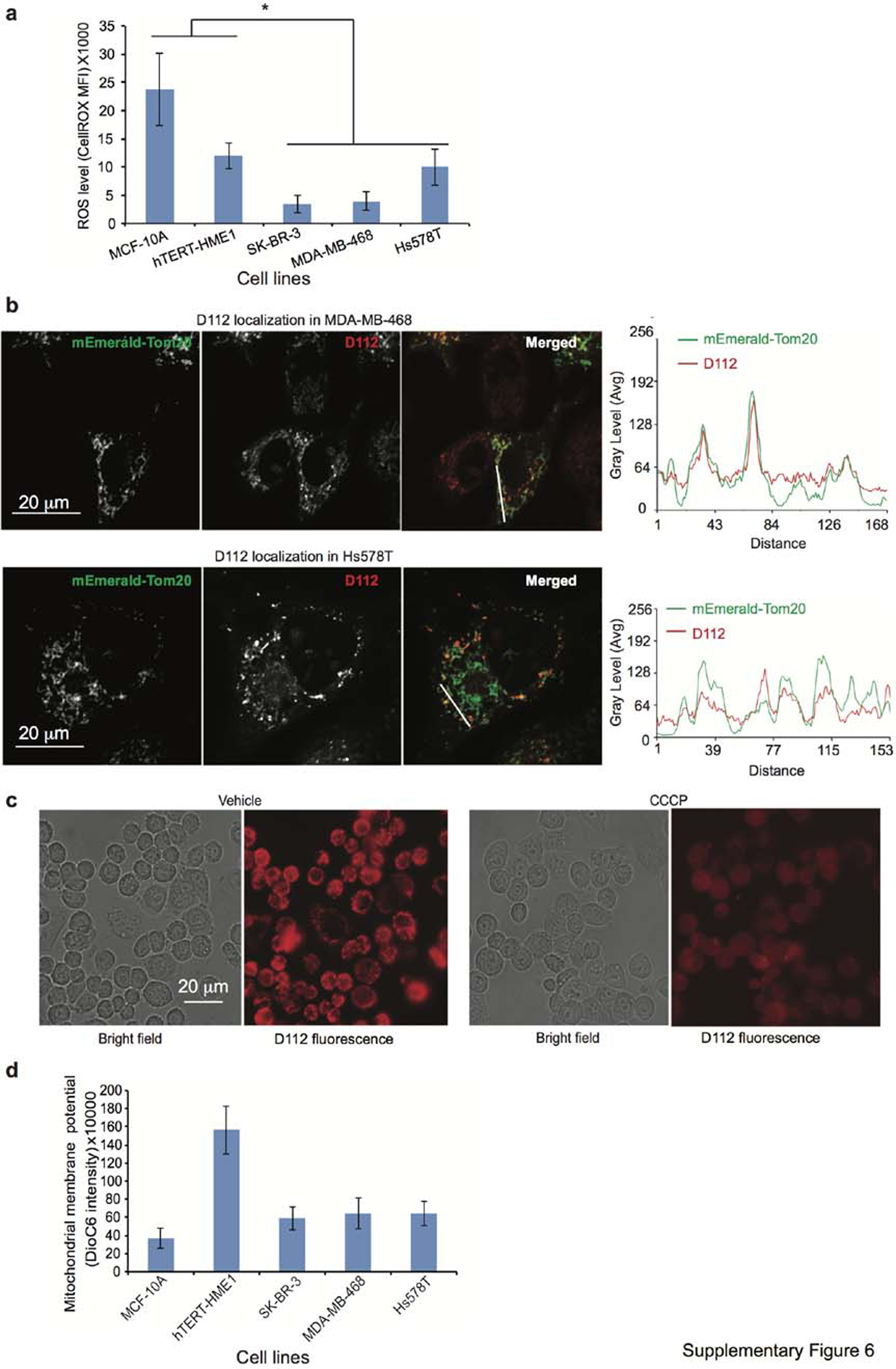

Supplement: Supplementary Figure 6 [file cddis201719x7.tif]

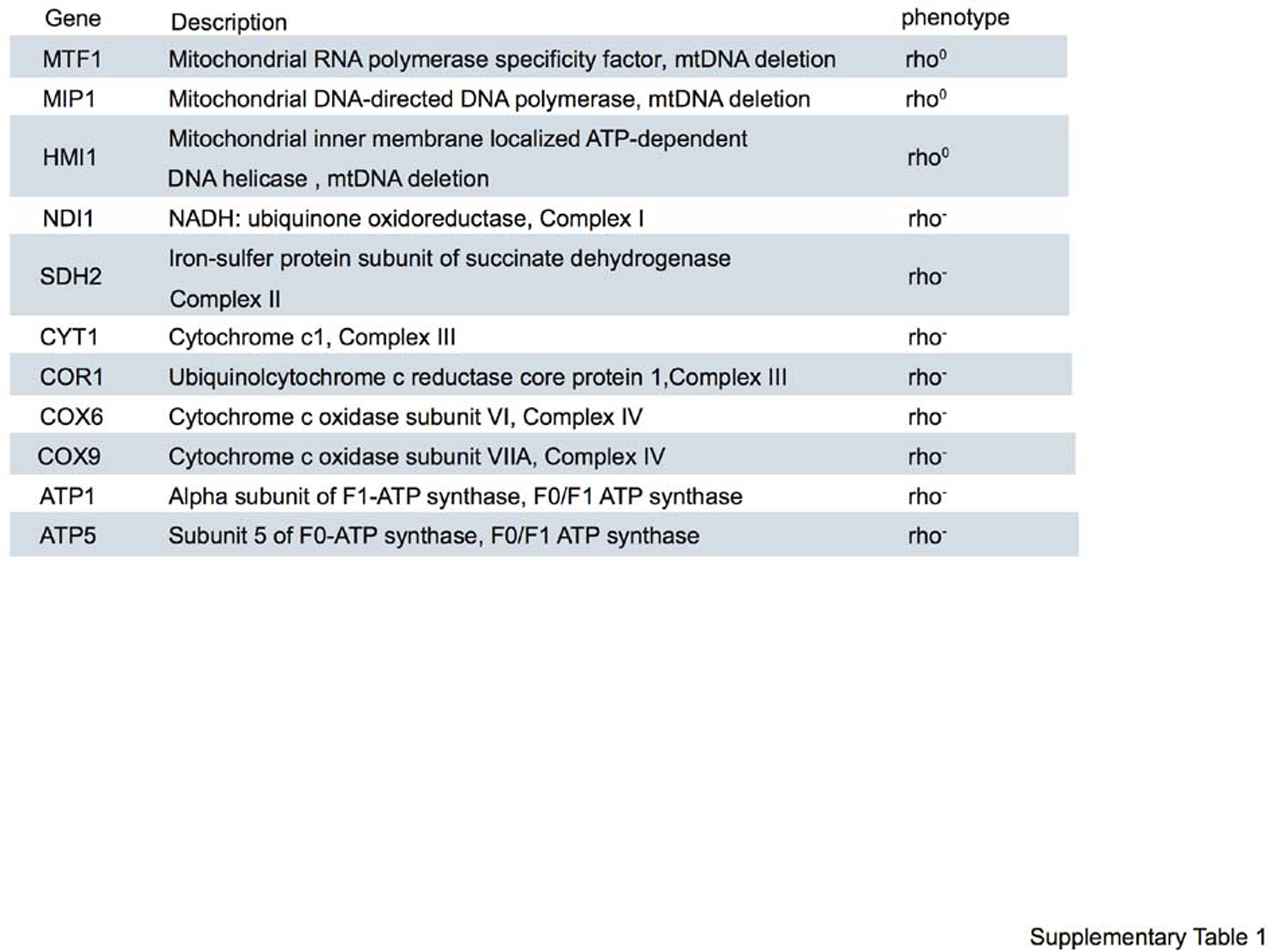

Supplement: Supplementary Table 1 [file cddis201719x8.tif]
